# Proteomics identification of overexpressed serum proteins in dogs with *Babesia* *canis* infection

**DOI:** 10.14202/vetworld.2023.2042-2048

**Published:** 2023-10-07

**Authors:** Sudpatchara Ritchoo, Phattara-orn Havanapan, Metita Sussadee, Cherdsak Maneeruttanarungroj, Rucksak Rucksaken

**Affiliations:** 1 Department of Veterinary Technology, Faculty of Veterinary Technology, Kasetsart University, Bangkok, Thailand; 2 Institute of Molecular Biosciences, Mahidol University, Salaya Campus, Nakhon Pathom, Thailand; 3 Department of Biology, School of Science, King Mongkut’s Institute of Technology Ladkrabang, Bangkok, Thailand

**Keywords:** *Babesia canis*, blood parasite, diagnosis, proteomics

## Abstract

**Background and Aim:**

Canine babesiosis, caused by the protozoan parasite *Babesia canis*, is characterized by clinical manifestations, including hemolytic anemia, thrombocytopenia, multiple organ failure, and may result in death. This disease is detected using conventional blood smears, which are time-consuming and have low sensitivity. This study aimed to investigate a more rapid and sensitive method for detecting *B. canis* infection in dogs by examining the expressed serum protein profiles using proteomics.

**Materials and Methods:**

We collected six sera samples from three healthy and three *B. canis*-infected dogs diagnosed using blood smear and polymerase chain reaction. We analyzed the proteins using two-dimensional gel electrophoresis. The candidate spots from the gel were subjected to protein identification using a nano-liquid chromatography system coupled to an ion-trap mass spectrometer equipped with an electrospray ionization nano-sprayer.

**Results:**

We found that 10 protein spots were overexpressed in the serum samples from infected dogs compared with healthy dogs, which corresponded to three proteins: serotransferrin, serotransferrin isoforms X1, and hemopexin. Furthermore, analysis of the protein-protein interaction network confirmed that they strongly interacted with each other.

**Conclusion:**

This study suggests that high levels of serotransferrin and hemopexin are related to *B. canis* infection, making these proteins potential candidates for the development of diagnostic molecules or vaccines.

## Introduction

Infection with *Babesia canis* causes canine babesiosis, a major animal health concern in tropical areas, including Thailand [1–4]. *Babesia* spp. are parasitic protozoa that live in vertebrates, such as cattle, pigs, horses, humans, and dogs [1]. *Rhipicephalus sanguineus*, or the brown dog tick, is an important vector for *Babesia* spp. [2, 5]. Clinical signs include hemolytic anemia, leucopenia, thrombocytopenia, and renal failure, occasionally leading to death [6, 7]. The *Babesia* spp. identified in Thailand includes *B. canis*, *Babesia vogeli*, and *Babesia gibsoni* [2, 3, 8]. In Thailand, the prevalence of *Babesia* spp.-infected dogs in the Khon Kaen province was 19.5% [2], *B. canis*-infected dogs in Maha Sarakham province was 6.3% [3], and *B. vogeli*-infected dogs in Songkhla and Narathiwat provinces was 8% [8]. Whereas the prevalence of *Babesia* spp. infection in the dogs treated at the Kasetsart University Veterinary Teaching Hospital, Bangkok, it was approximately 20% [9]. At present, *Babesia* spp. infections are diagnosed by microscopic examination of blood smears. However, this method has low sensitivity and may not detect the pathogen in cases where the infection level is low in the blood sample or chronic infections [10]. Although the polymerase chain reaction (PCR) assay is a more sensitive and efficient method to identify pathogens [11], it requires expensive equipment, experienced staff, and a long processing time. Indirect fluorescent antibody test is a technique used to detect antibodies (immunoglobulin (Ig)M and IgG titers), but it has low specificity as it cannot distinguish between treated and infected dogs [12].

A new mass spectrometry (MS)-based approach has been developed to identify blood parasites and their protein markers [7, 13]. Mass spectrometry offers several advantages, including high sensitivity, specificity, and the ability to rapidly and simultaneously analyze multiple compounds [14]. Overexpressed proteins can potentially be used for diagnosis, disease monitoring, and prediction. During infection, the host’s defense mechanisms respond by altering protein production. As these proteins are secreted into the blood circulation, their expression levels in blood samples can be used to distinguish between healthy and infected animals [15–17]. The proteins generated during infection can be used as biological markers. Studies have shown that the levels of certain proteins increase during specific diseases, making them ideal diagnostic tools [18–20]. Proteomics is a highly effective *in vitro* technique that involves protein separation, detection, and identification. Therefore, this approach can be used to understand disease initiation and response and to develop advanced diagnostic tools, medications, and vaccines [16, 21].

Thus, in this study, we analyzed the changes in serum proteins in *B. canis*-infected dogs using proteomics.

## Materials and Methods

### Ethical approval

The research protocol of this study was approved by the Animal Ethics Committee of the Faculty of Veterinary Technology, Kasetsart University, Thailand (ACKU59-VTN-003).

### Study period and location

This study was conducted from April 2017 to March 2018 at the Institute of Molecular Biosciences, Mahidol University, and the Faculty of Veterinary Technology, Kasetsart University. The blood samples were obtained from Faculty of Veterinary Technology, Kasetsart University, Bangkok, Thailand, and Khon Kaen University Veterinary Hospital, Khon Kaen, Thailand.

### Animals

We collected 2 mL of whole blood samples from three healthy and three naturally infected dogs. Sera were stored at −20°C until analysis. All the dogs were male, with a mean age of 61 ± 8 months. In addition to physical examinations, all dogs underwent blood tests to detect *B. canis* using blood smears. The infected dogs were also isolated by species to confirm *B. canis* infection using a PCR assay. The healthy dogs did not have any history of infections with *Babesia* spp. or other blood parasites.

### Polymerase chain reaction assay

Polymerase chain reaction was performed as described previously by Rucksaken *et al*. [3]. Briefly, a 25 μL reaction was prepared containing 1× DreamTaq Green buffer (Thermo Fisher Scientific, Waltham, MA, USA), 0.2 mM dNTPs, 1 μM PCR primer, 1 μL DNA template, and 1.25 units of DreamTaq DNA polymerase (Thermo Fisher Scientific). Ultrapure sterile water was added to make up the volume to 25 μL. The PCR cycles consisted of initial denaturation at 95°C for 2 min, denaturation at 95°C for 30 s, annealing at 55°C for 30 s, and extension at 72°C for 30 s, for 35 cycles. The PCR products were visualized using electrophoresis on 1.5% agarose gel with SYBR Safe DNA gel stain (Thermo Fisher Scientific) under ultraviolet light. The positive blood samples were used as positive controls.

### Two-dimensional electrophoresis (2-DE)

Albumin in the sera from healthy and infected dogs was eliminated using the Albumin/IgG Removal Kit (Thermo Fisher Scientific). After cleaning up the samples using a 2-D Clean-Up Kit (GE Healthcare, Chicago, IL, USA), the total protein concentration was measured using the Bradford assay. Then, 150 μg of serum protein was separated based on isoelectric focusing. Immobilized pH gradient strips (7 cm in length with a non-linear gradient pH 3–10) were used. Ettan IPGphor II (GE Healthcare) was used for protein separation in the first dimension with a focusing profile that increased the voltage to 8720 V/h for 15 h at 22°C. Subsequently, the strips were equilibrated using 1% (w/v) dithiothreitol in the equilibrium buffer for 15 min and 2.5% (w/v) iodoacetamide in the equilibrium buffer for 15 min. The second dimension was performed using 12.5% sodium dodecyl sulfate-polyacrylamide gel electrophoresis (SDS-PAGE). A 2% agarose gel with bromophenol blue was added before protein separation using a constant 0.04 A current at 25°C. The gels were then stained with Coomassie brilliant blue G250 before being destained. The gels were captured using the Image scanner II (GE Healthcare) and the differential protein spots were analyzed using the SameSpots software V.5.1 (TotalLab, Newcastle, UK). Finally, we selected ten overexpressed protein spots with ≥1.2-fold change, which were trypsin-digested and submitted for protein identification using a nano-liquid chromatography system coupled to an ion-trap mass spectrometer equipped with an ESI nano-sprayer.

### Mass spectrometry

The MS data were used to identify the proteins using the MASCOT MS/MS Ion Search tool (Matrixscience, London, UK) and the NCBIprot (Mammalia [mammals]) database. The parameters for the Mascot search were as follows: Enzyme: trypsin; carbamidomethylation (C) as a fixed modification; oxidation (HW), and oxidation (M) as variable modifications; peptide mass tolerance of 0.5 Da and fragment mass tolerance of 0.5 Da; a peptide charge state of +1, +2, +3; instrument type: Electrospray ionization (ESI)-TRAP; and report top: auto. A MASCOT score >16 indicated a significant match (p < 0.05) with a known protein.

### Protein-protein interaction network analysis

The protein-protein interactions were analyzed using the STRING database version 11.5 (https://string-db.org). Interaction network analysis was performed using proteins from the *Canis lupus* species. The interactions were assigned a medium confidence score of 0.4.

### Statistical analysis

The protein expression levels and relative band intensities of healthy and *B. canis*-infected dogs were compared using analysis of variance. p < 0.05 was considered statistically significant. Statistical analysis was performed using SameSpots software V.5.1 (https://totallab.com/products/samespots/).

## Results

**Figure-1:**
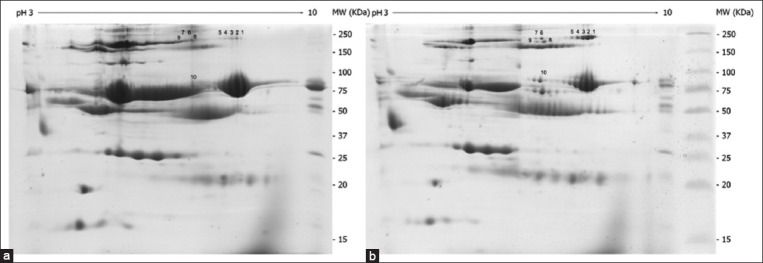
Representative of (a) two-dimensional electrophoresis analysis of healthy and (b) *Babesia canis*-infected dog sera.

**Figure-2:**
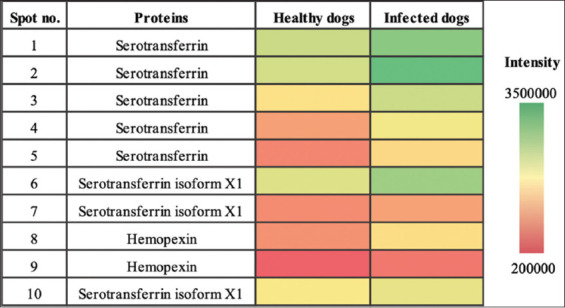
Heatmap showing the relative abundance (color) of 10 candidate proteins.

**Figure-3:**
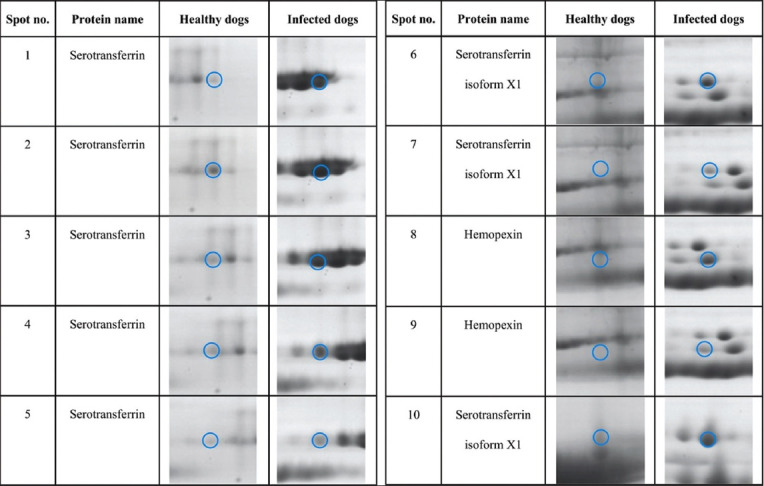
Different protein spots (numbered 1–10) in sera of dogs infected with *Babesia canis* compared with healthy dogs’ sera.

We used the 2-DE proteomic approach to identify and compare the changes in the serum protein levels in *B. canis*-infected and healthy dogs. The serum protein profiles identified were different for each group (Figure-1). The gel images revealed 10 protein spots showing higher expression in infected dogs than healthy dogs (Figure-2). We subjected these protein spots to protein identification using a nano-liquid chromatography system coupled to an ion-trap mass spectrometer equipped with an ESI nano-sprayer (Figure-3).

Based on the MS results, we identified three proteins from the 10 protein spots, namely, serotransferrin from spots 1–5 (p *=* 0.811, 0.730, 0.655, 0.408, and 0.357, respectively), serotransferrin isoform X1 from spots 6, 7, and 10 (p *=* 0.729, 0.655, and 0.964, respectively), and hemopexin from spots 8–9 (p = 0.502 and 0.703, respectively) (Table-1).

**Table-1:** Identification of 10 candidate protein spots from healthy and infected dogs using mass spectrometry.

Spot no.	Protein name	Protein score	Fold change	Mass (Da)	Protein matches	Species	Sequence	Function
1-5	Serotransferrin	1347	1.4-2.2	80222	26 (26)	*Canis lupus familiaris*	K.VPSHAVVAR.S	Iron binding, acute-phase response
6-7	Serotransferrin isoform X1	1621	1.5-2.2	79136	55 (14)	*Canis lupus familiaris*	K.NTEDWAK.D	Iron binding, acute-phase response
8-9	Hemopexin	627	1.7-2	52047	18 (4)	*Canis lupus familiaris*	K.SLPQPQR.V	Binding protein
10	Serotransferrin isoform X1	1798	1.2	79136	61 (17)	*Canis lupus familiaris*	K.NPEAWAK.D	Iron binding, acute-phase response

**Figure-4:**
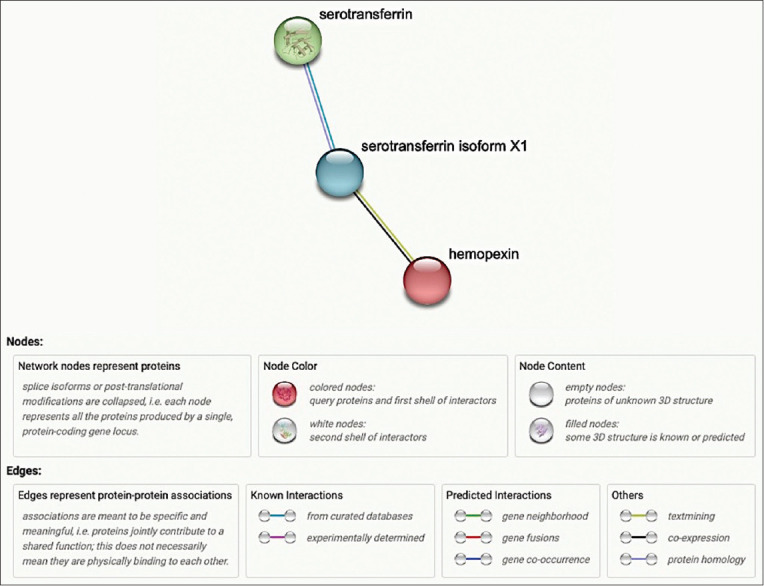
Protein-protein interactions and statistical significance of candidate proteins based on STRING analysis.

The protein-protein interaction analysis indicated that these proteins (serotransferrin, serotransferrin isoform X1, and hemopexin) strongly interact with each other. The data also shows coexpression of serotransferrin isoform X1 and hemopexin. The thickness of the lines connecting the proteins in Figure-4 represents the strength of their interaction based on the supporting data.

## Discussion

The serum protein profiles of dogs infected with *B. canis* are altered [16]. Proteomics is a useful tool for searching and separating target proteins from samples, including sera and blood. This is the first report on a proteomics analysis of blood samples from *B. canis*-infected dogs in Thailand. In this study, we used MS analysis to compare the serum protein profiles of healthy and *B. canis*-infected dogs to identify potential disease biomarkers. Our MS analysis revealed significant differences between both groups in several serum protein spots. Particularly, the levels of serotransferrin, serotransferrin isoform X1, and hemopexin were elevated in the infected dogs. The previous study by Kuleš *et al*. [16] showed that increased expression levels of serum proteins, including serotransferrin and hemopexin, are involved in inflammation-mediated, acute-phase response, complement, and coagulation cascades, apolipoproteins, and Vitamin D metabolism pathways in *B. canis*-infected dogs. Moreover, serotransferrin and serotransferrin isoform 6 were discovered in mice infected with *Babesia microti* at 11 days after infection and in dogs infected with *Dirofilaria immitis*, respectively [22, 23]. These proteins are involved in hemoglobin metabolism during hemolysis and acute inflammation during blood parasite infection [16].

Serotransferrin, or serum transferrin (Trf), is a serum glycoprotein consisting of 679 amino acids [24] and has several isoforms [25]. It is essential for iron binding, inflammatory response, and antimicrobial activity [16, 22, 26]. It binds and transports iron from the small intestines or dead erythrocytes to target tissues, such as bone marrow, for hemoglobin production [26, 27]. Alexander-Bryant *et al*. [28] reported increased Trf receptors in inflammatory or cancer cells, indicating that the serotransferrin levels might increase during inflammation or cancer. Serotransferrin participates in the host defense mechanism by binding to free iron ions in hosts so that pathogens do not get enough iron to proliferate [22]. Primarily, serotransferrin is expressed in the liver [27], with minor expression in the placenta, kidney, and mammary gland [26, 29, 30]. Moreover, Trf has been associated with enhanced therapeutic efficacy in cancer [31, 32].

Hemopexin, also known as beta-1B-glycoprotein [33], is a vital acute-phase response protein involved in several physiological and pathological processes [34]. Hemopexin has the highest affinity for heme (K_d_ <1 pmol), allowing it to bind and transport a substantial quantity of free heme in plasma [35]. Hemopexin is expressed mainly in the hepatic parenchymal cells, with minor expression in the neurons and astrocytes of the central nervous system, ganglionic and photoreceptor cells of the retina, Schwann and fibroblast-like cells of the peripheral nervous system, kidney mesangial cells, skeletal muscles, and ovaries [35, 36]. Hemopexin binds to the heme released into the plasma after hemolysis or tissue injury and transports it to the liver for breakdown and iron recovery before returning into circulation as free hemopexin. In addition, the heme-scavenging activity of hemopexin prevents oxidative damage caused by free heme [37]. Furthermore, hemopexin can bind nitric oxide (NO) and carbon monoxide and protect against NO-mediated toxicity, notably during trauma and hemolysis, which are pathological features of *B. canis* infection [35].

Serotransferrin and hemopexin are implicated in the pathological lesions resulting from *B. canis* infection. While functional analysis of these proteins suggests that they can be potential diagnostic biomarkers or vaccine candidates for canine babesiosis, large-scale studies and further validation using techniques, such as immunoblotting or enzyme-linked immunosorbent assay, are required.

## Conclusion

Using proteomics, we examined the serum protein profiles of *B. canis*-infected dogs and found that these dogs had higher serum levels of hemopexin and serotransferrin. These proteins are promising disease biomarkers or vaccine candidates for canine babesiosis, although their efficacy requires further validation.

## Authors’ Contributions

SR: Investigation and writing-original draft preparation. PH: Methodology and editing of the manuscript. MS: Data analysis and editing of the manuscript. CM: Conceptualization, reviewing and editing of the manuscript. RR: Supervision, conceptualization, writing-reviewing, and editing of the manuscript. All authors have read, reviewed, and approved the final manuscript.
